# Calycosin protects against chronic prostatitis in rats via inhibition of the p38MAPK/NF-κB pathway

**DOI:** 10.1515/med-2023-0770

**Published:** 2023-08-28

**Authors:** Heng Wang, Lei He, Zhaofei Liu, Xiangjun Xu, Haitao Zhang, Pengfei Mao, Ming Li

**Affiliations:** Department of Urology, Lianyungang TCM Hospital Affiliated to Nanjing University of Chinese Medicine, Lianyungang 222000, China; Department of Acupuncture, Lianyungang TCM Hospital Affiliated to Nanjing University of Chinese Medicine, Lianyungang 222000, China; Department of Pharmacy, Lianyungang TCM Hospital Affiliated to Nanjing University of Chinese Medicine, No.160 Chaoyang Middle Road, Haizhou District, Lianyungang 222000, China

**Keywords:** calycosin, chronic prostatitis, p38MAPK/NF-κB pathway

## Abstract

Currently, the effect and molecular mechanism of calycosin, the main active ingredient of Qinshi Simiao San, which can alleviate chronic prostatitis (CP), on CP remain unclear. This study aimed to elucidate the potential mechanism of action of calycosin in CP in a rat CP model. The prostate tissue morphology was evaluated based on hematoxylin–eosin staining. Enzyme-linked immunosorbent assay was conducted to evaluate inflammatory cytokine and immune factor levels (secretory immunoglobulin A [SIgA]; immunoglobulin G [IgG]) in prostate tissues and serum. Additionally, representative biomarkers of oxidative stress, including malondialdehyde, superoxide dismutase, and catalase were detected using detection kits, and reactive oxygen species release was evaluated using immunofluorescence staining. Furthermore, the p38 mitogen-activated protein kinase (p38MAPK)/NF-kappaB (NF-κB) signaling pathway was analyzed by western blotting. The results showed that calycosin substantially ameliorated the pathological damage to prostate tissues of the CP rats. Moreover, calycosin significantly downregulated interleukin (IL)-1β, IL-6, and tumor necrosis factor-alpha, IgG, and SIgA levels. Furthermore, we found that calycosin considerably suppressed oxidative stress and inhibited the activation of the p38MAPK/NF-κB signaling pathway in rats with CP. In summary, our findings revealed that calycosin protects against CP in rats by inhibiting the p38MAPK/NF-κB pathway.

## 1 Introduction

Chronic prostatitis (CP) is a common disease of the urinary system with a high incidence rate that seriously affects the quality of life of patients. The symptoms of CP are complex and diverse and include abnormal urination, inguinal pain, insomnia, and severe infertility [1,2]. Thermobalancing therapy [3], acupuncture therapy [4], and medication are commonly used to treat CP; however, their long-term effects are unsatisfactory. Thus, timely diagnosis and effective treatment strategies for CP have become popular research topics.

An increasing number of studies have reported that abnormal immune responses, inflammation, neuroendocrine abnormalities, and oxidative stress-induced damage are closely associated with CP progression. Lin et al. confirmed that targeting ferroptosis suppressed inflammation, fibrosis, and mast cell activation in CP [5]. Furthermore, Zhao et al. demonstrated that lycopene attenuates CP by suppressing oxidative stress and inflammation through the regulation of the NF-kappaB (NF-κb), mitogen-activated protein kinase (MAPK), and Nrf2 signaling pathways in rats [6]. The p38MAPK and NF-κB signaling pathways are also linked to inflammation in CP; Meng et al. reported that quercetin protects against CP in a rat model by acting on the NF-κB and MAPK signaling pathways [7]. Therefore, regulating the p38MAPK/NF-κB signaling pathway may be a novel strategy for blocking CP development by inhibiting inflammation.

Qinshi Simiao San (QSSMS) is the most frequently prescribed medication for the treatment of CP [8]. Previous studies have shown that the active ingredients of QSSMS are quercetin, kaempferol, formononetin, isorhamnetin, and calycosin [8,9]. Calycosin, an isoflavone, is a vital regulator of multiple diseases. Zhang et al. found that calycosin inhibited breast cancer cell migration and invasion by suppressing epithelial-mesenchymal transition via BATF/TGF-beta1 [10]. Moreover, Hu et al. found that calycosin inhibited autophagy and oxidative stress in chronic kidney disease skeletal muscle atrophy by regulating the AMPK/SKP2/CARM1 signaling pathway [11]. However, the role and molecular mechanisms of calycosin in CP require further investigation.

In the present study, we aimed to elucidate the latent mechanism of calycosin in CP and to verify our conclusions in a rat model.

## 2 Materials and methods

### 2.1 Animals

Male Wistar rats (6–8 weeks old, 210 ± 10 g) were purchased from Vital River Laboratory Animal Technology Co., Ltd. and cultured under constant conditions (23 ± 1℃, 60 ± 5% humidity, 12 h light/dark cycle).


**Ethics approval and consent to participate:** The experimental protocols were approved by Ethical Committee of the Experimental Animal Center of Lianyungang TCM Hospital Affiliated to Nanjing University of Chinese Medicine according to the National Institutes of Health Guide for the Care and Use of Laboratory Animals.

### 2.2 Establishment of CP model and treatment

Fifty rats were divided into five groups: control, model, model + calycosin (10 mg/kg), model + calycosin (20 mg/kg), and model + calycosin (30 mg/kg) [12,13]. The CP rat model was established according to the method described by Wang et al. [14]. After the rats were anesthetized with ether, 1 cm of the abdominal skin was cut to expose the prostate. The rats were then injected with 3% carrageenan into the left and right ventral lobes of the prostate to establish the CP model, and 0.9% sodium chloride solution was injected into the control group. After 7 days, high, medium, and low doses of calycosin were administered by gavage. After 28 days of administration, the prostate tissues of the rats in each group were removed and the rats were sacrificed by peeling off their necks.

### 2.3 Evaluation of inflammation scores

Two samples were collected at the beginning and end of RNA isolation, and each tissue slice was evaluated for inflammation according to an effective four-stage grading system: no inflammation (0), mild inflammation (1), moderate inflammation (2), and severe inflammation (3). Mild inflammation was characterized by small and confluent aggregations of inflammatory cells. Moderate inflammation was defined as the presence of larger multifocal aggregates visible at low magnification. Severe inflammation was defined as large, extensive, and confluent aggregates evident at low magnification. The scores of the two samples were summed to obtain total scores ranging from 0 to 6.

### 2.4 Detection of immunoglobulin (Ig)

Secretory immunoglobulin A (SIgA) (ELK1548; ELK Biotechnology) levels in the prostate tissue and IgG (ELK1254; ELK Biotechnology) levels in the serum of rats were detected using enzyme-linked immunosorbent assay (ELISA) kits following the manufacturer’s instructions. The optical density of each well was measured at 450 nm using a Multiscan Spectrum (MD, USA) according to the manufacturer’s instructions.

### 2.5 Hematoxylin–eosin (HE) staining

After the rat prostate tissue was fixed with 4% paraformaldehyde for more than 24 h and dehydrated, 2–3 μm paraffin sections were taken from the fixed prostate tissue. The sections were then dewaxed, hydrated, subjected to hematoxylin staining for 3–10 min, and stained with eosin for 1–3 min according to the manufacturer’s instructions. The morphology of the prostate tissue was observed under a microscope.

### 2.6 Determination of ELISA

After treatment, blood was drawn from the rats and centrifuged, and serum was collected. The prostate tissue was thoroughly ground using a homogenizer and centrifuged, and the supernatant was collected. The samples were cultured in a 96-well plate and HRP-conjugate reagent was added to each well. Subsequently, the levels of inflammatory factors interleukin (IL)-1β (ELK1272; ELK Biotechnology); IL-6 (ELK1158; ELK Biotechnology); and tumor necrosis factor-alpha (TNF-α) (ELK1396; ELK Biotechnology) in the samples were determined using ELISA kits following the manufacturer’s instructions. The optical density of each well at 450 nm was measured using a Multiscan Spectrum according to the manufacturer’s instructions.

### 2.7 Measurement of malondialdehyde (MDA), superoxide dismutase (SOD), and catalase (CAT) levels

After treatment, SOD (A001-3), CAT (A007-1), and MDA (A003-1) levels in the prostate tissue were determined using the Total SOD Assay Kit, CAT Assay Kit, and Lipid Peroxidation Assay Kit (Nanjing Jiancheng Bioengineering Institute, Nanjing, China), respectively, according to the manufacturer’s instructions.

### 2.8 Measurement of reactive oxygen species (ROS)

After the prostate tissue sections were routinely dewaxed and treated with a 0.3% H_2_O_2_ methanol solution to inhibit endogenous peroxidase, DHE-ROS fluorescent probes (KGAF019; KeyGEN Biotech, Nanjing, China) were added and nucleated with DAPI (D8417-1MG; Sigma-Aldrich, St. Louis, MO, USA) for 3 min; the slices were then sealed, observed, and photographed under a laser scanning confocal microscope.

### 2.9 Western blot assay

Prostate tissues were lysed using RIPA buffer (AS1004; ASPEN) for 30 min. Proteins were resolved using an SDS-PAGE kit (AS1012; ASPEN) and transferred onto PVDF membranes (IPVH00010; Millipore). The membranes were blocked with 5% skimmed milk for 2 h to avoid nonspecific binding and then cultivated with primary antibodies against p-p38 (#4511, 1:500; Cell Signaling Technology, Danvers, MA, USA), p38 (#8690, 1:5,000; Cell Signaling Technology), p-p65 (#3033, 1:500; Cell Signaling Technology), p65 (#8242, 1:5,000; Cell Signaling Technology), or β-actin (TDY051, 1:10,000; Beijing Tad Biotech Co., Ltd., Beijing, China) at 4℃ overnight. After washing thrice with TBST, the membranes were incubated with secondary antibodies for 2 h. The protein signals were assessed using the electrochemiluminescence method (AS1059; ASPEN) following the manufacturer’s instructions, and the data were analyzed using ImageJ software version 1.8.0 (NIH, Bethesda, MD, USA).

### 2.10 Statistical analysis

SPSS v.19.0 (IBM Corp., Armonk, NY, USA) was used for statistical analysis. Results are presented as mean ± standard deviation from three independent experiments. Differences among groups were evaluated using a one-way analysis of variance or Student’s *t*-test. *P < 0.05 and **P < 0.01 were considered statistically significant.

## 3 Results

### 3.1 Calycosin significantly relieved pathological injury in CP rats

**Figure 1:**
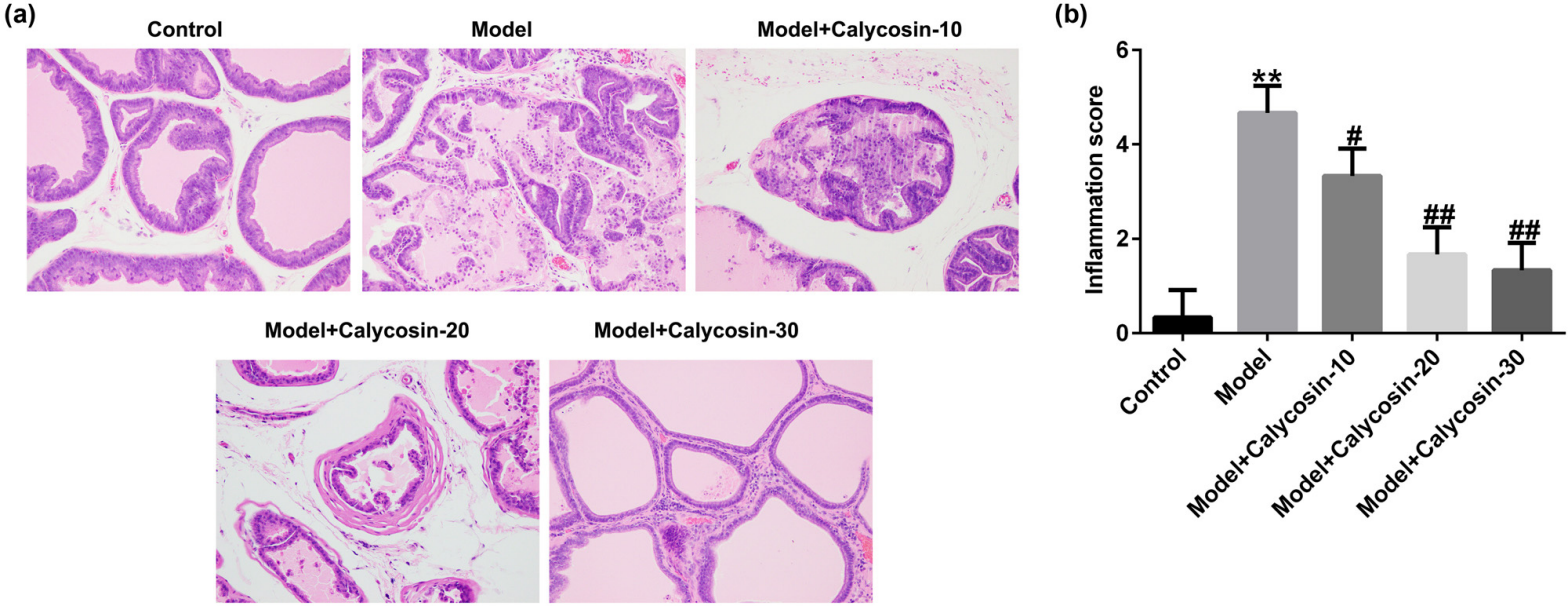
HE staining of prostate tissues in different groups. (a) HE staining image of rat prostate tissue. (b) Inflammation score of prostate tissue in different groups. **P < 0.01 vs control; ^#,##^ *P* < 0.05, 0.01 vs model.

We evaluated the morphology of the prostate tissues in each group using HE staining. As shown in Figure 1a, the rats showed apparent lymphocyte infiltration, decreased acinar diameter, glandular cavity expansion, and interstitial edema 7 days after 3% carrageenan injection, which indicated the successful establishment of the CP rat model. After calycosin administration for 28 days, the glandular cavity structure of the prostate tissues recovered; the interstitial space, inflammatory cell infiltration in the glandular cavity and interstitial space, and migration of fibroblasts and blood vessels decreased in a dose-dependent manner. Specifically, the improvement was most significant in the 30 mg/kg calycosin group. These findings confirmed that calycosin can ameliorate CP. Furthermore, the inflammatory response was scored in the prostate tissues, and we found that the inflammation score of the prostate tissue remarkably increased in the model group compared to the control group (Figure 1b). However, this effect was reduced by calycosin treatment in a dose-dependent manner. Our data demonstrated that calycosin significantly mitigated the pathological damage in rats with CP.

### 3.2 Calycosin notably inhibited inflammatory response in rats with CP

**Figure 2:**
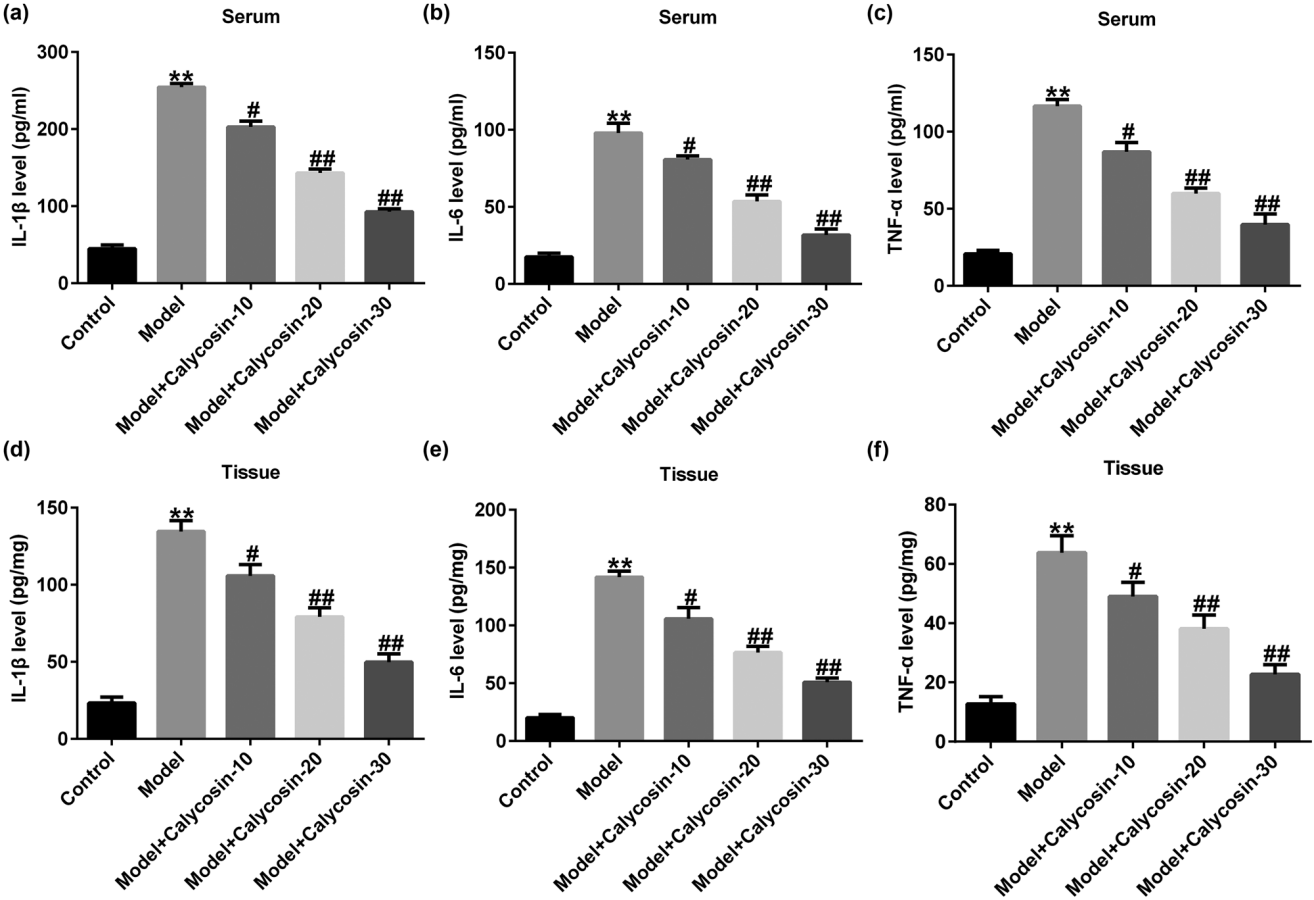
Effects of calycosin on inflammatory response in prostate tissues. Serum levels of IL-1β (a), IL-6 (b), and TNF-α (c) were analyzed by ELISA. IL-1β (d), IL-6 (e), and TNF-α (f) expression levels were detected in the prostate tissue. **P < 0.01 vs control; ^#,##^ *P* < 0.05, 0.01 vs model.

Immune reactions and oxidative stress are key indicators of CP [15]. We analyzed the secretion of inflammatory cytokines (IL-1β, IL-6, and TNF-α) in rat serum and prostate tissues using ELISA. As shown in Figure 2, the levels of IL-1β, IL-6, and TNF-α in rat serum (Figure 2a–c) and prostate tissue (Figure 2d–f) were higher in the model group than in the control group. After calycosin treatment, the expression of inflammation-related factors decreased in a dose-dependent manner. In summary, these data indicated that calycosin repressed inflammatory responses in rat prostate tissues.

### 3.3 Calycosin substantially reduced the expression of Ig in rats with CP

**Figure 3:**
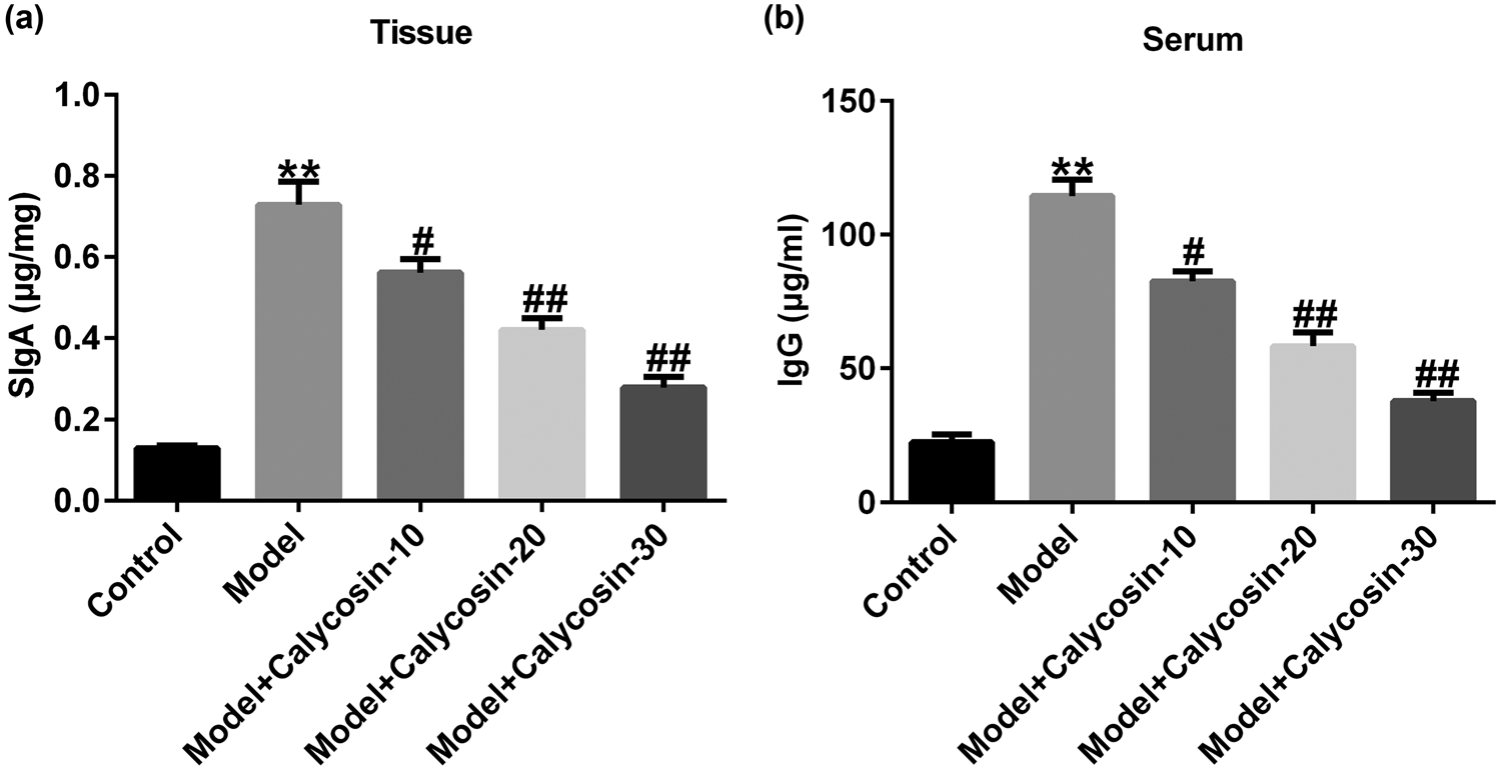
Effects of calycosin on Ig in chronic prostatitis. ELISA was used to detect Ig levels in rats with CP, including SIgA levels (a) in prostate tissue and IgG levels (b) in rat serum. **P < 0.01 vs control; ^#,##^ *P* < 0.05, 0.01 vs model.

We measured the levels of immune factors in rats with CP. In the model group, the SIgA levels and IgG secretion in sera of rats with CP increased, whereas these changes were reversed in a dose-dependent manner after calycosin treatment (Figure 3a and b). Our data revealed that calycosin reduced the levels of immune factors in rats with CP.

### 3.4 Calycosin notably suppressed oxidative stress in rats with CP

**Figure 4:**
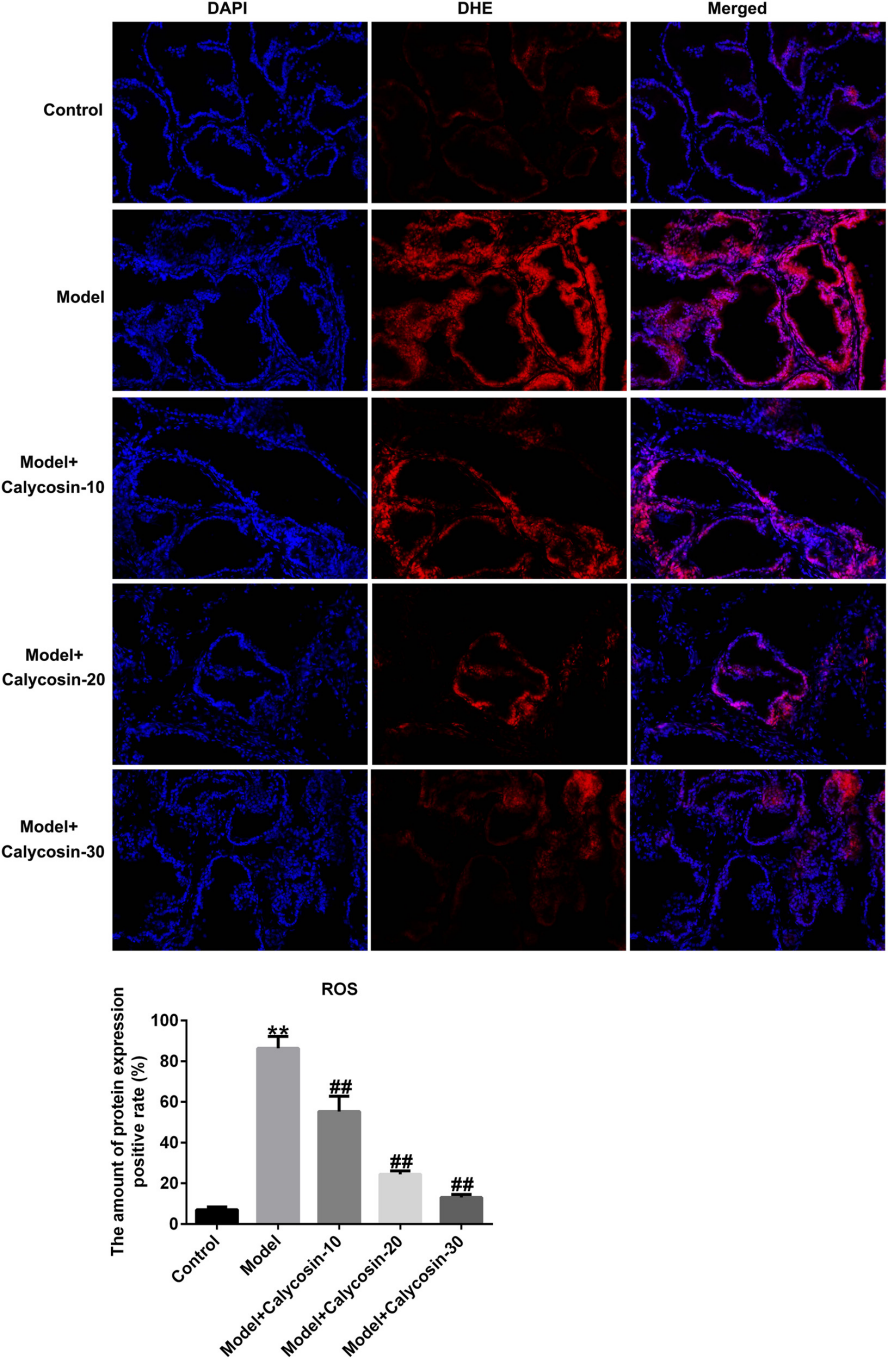
Effects of calycosin on ROS release in chronic prostatitis. Immunofluorescence experiments were performed to determine ROS release in prostate tissues. **P < 0.01 vs control; ^##^ *P* < 0.01 vs model.

**Figure 5:**
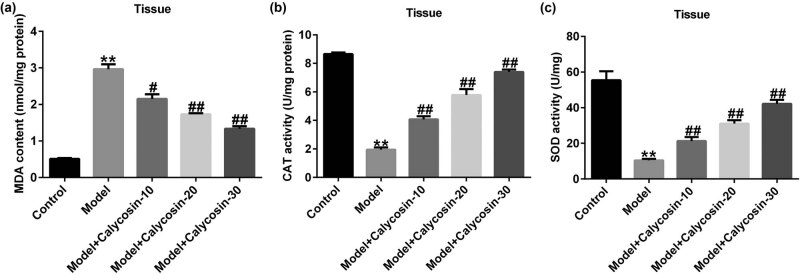
Effects of calycosin on oxidative stress in chronic prostatitis. Expression of MDA (a), CAT (b), and SOD (c) in prostate tissues. **P < 0.01 vs control; ^#,##^ *P* < 0.05, 0.01 vs model.

Abnormal oxidative stress can also cause prostate tissue damage. Based on previous investigations, we determined whether calycosin functions in oxidative stress in CP by measuring ROS, MDA, SOD, and CAT levels. Compared to the control group, we observed higher ROS release (Figure 4) and MDA levels (Figure 5a) as well as lower CAT and SOD activities (Figure 5b and c) in the prostate tissue. However, the opposite trend in the calycosin treatment group in a dose-dependent manner, suggesting that calycosin suppressed oxidative stress and inflammation in rats with CP.

### 3.5 Calycosin significantly inhibited the activation of the p38MAPK/NF-κB signaling pathway in rat prostate tissue

**Figure 6:**
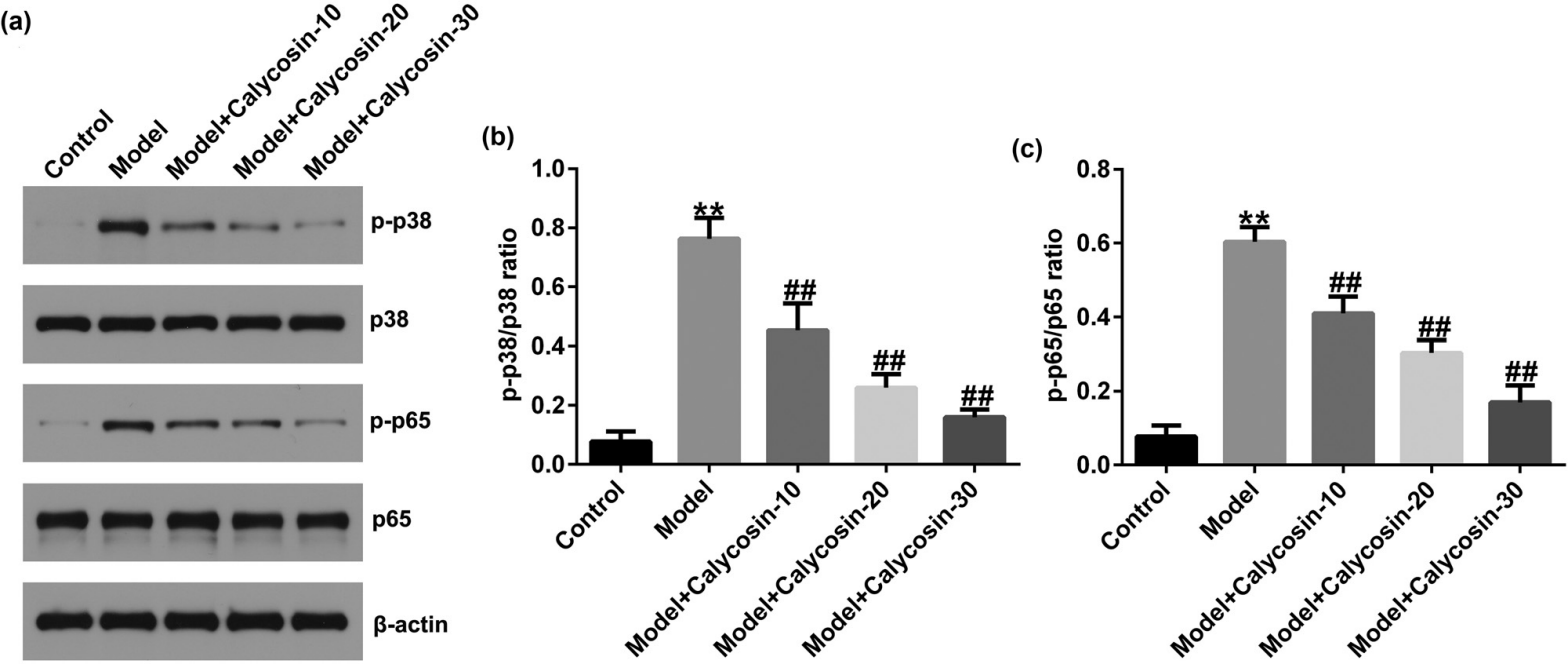
Effect of calycosin on p38MAPK/NF-κB pathway in prostate tissues. (a) Western blot analysis of p-p38, p38, p-p65, and p65 expression in prostate tissue. (b and c) Determination of p-p38/p38 ratio or p-p65/p65 value. **P < 0.01 vs control; ^##^ *P* < 0.01 vs model.

The NF-κB and p38MAPK signaling pathways were found to be related to inflammation [16]. Thus, we further examined the molecular mechanism of calycosin in the p38MAPK/NF-κB signaling pathway in the prostate tissue of rats. As shown in Figure 6a–c, calycosin reversed the expression of p-p38 and p-p65 in the model group in a dose-dependent manner, as confirmed by the suppression of p-p38 and p-p65 levels and decreased p-p38/p38 and p-p65/p65 ratios. Our findings revealed that calycosin protects against CP in rats by inhibiting the p38MAPK/NF-κB pathway.

## 4 Discussion

The findings of the current study indicated that calycosin relieves CP by regulating inflammatory responses and oxidative stress through the inhibition of the p38MAPK/NF-κB signaling pathway, which may provide a theoretical foundation for CP therapy.

CP mainly occurs in middle-aged men and is characterized by chronic pain or discomfort in the perineum or lower abdomen. The pathogenesis of CP is complex and its symptoms frequently recur [17,18]. Drugs can temporarily relieve some symptoms; however, no effective treatment is currently available. Therefore, exploring effective measures to prevent CP and identifying effective treatment strategies have become a major focus of current research. QSSMS is a well-known traditional Chinese medicine that has been proven to significantly ameliorate CP. The bioactive components of QSSMS are quercetin, kaempferol, formononetin, isorhamnetin, and calycosin [8,9]. Calycosin is an isoflavone involved in the regulation of oxidation–reduction, immune, and inflammatory responses [19]. However, the role and molecular mechanisms of calycosin in CP remain unclear. Therefore, this study aimed to explore the role of calycosin in a rat model of CP and clarify its molecular regulatory mechanisms.

We first established a rat model of CP by injecting 3% carrageenan into the left and right ventral lobes of the SD rat prostate. On the seventh day after carrageenan injection, we found that the pathological changes were similar to those observed in the model developed by Li et al. [8], with apparent lymphocyte infiltration, decreased acinar diameter, glandular lumen expansion, and interstitial edema, indicating that the CP rat model had been successfully established. However, after 28 days of calycosin (low, medium, and high concentrations) stimulation, we observed the following: the gland cavity structure of the prostate tissue was significantly repaired, inflammatory cell infiltration was reduced, the interstitial space was narrowed, and the proliferation of fibroblasts and blood vessels was reduced in a dose-dependent manner, especially in the high-dose group. We also found that the inflammation score of the prostate tissue was remarkably higher in the model group than in the control group. However, this effect was reduced by calycosin in a dose-dependent manner, suggesting that calycosin remarkably mitigated the pathological damage in rats with CP.

Studies have shown that inflammation, abnormal immune responses, oxidative stress injury, and endocrine dysfunction are closely related to CP occurrence [20]. The increased expression of the pro-inflammatory mediators TNF-α, IL-1β, IL-6, and IL-8 is the main cause of CP [21]. Our research showed that IL-1β, IL-6, and TNF-α expression was higher in the model group than in the control group, which is consistent with the findings of Wang et al. [21]. However, the levels of IL-1β, IL-6, and TNF-α decreased significantly after calycosin treatment, which confirmed that calycosin could inhibit inflammation. High levels of IgG in the serum of patients with CP may be an essential factor in its pathogenesis. The immune response of serum IgG to SP protein in patients with CP was remarkably upregulated, which may lead to inflammation and male reproductive tract cell infiltration [21]. Therefore, we analyzed the levels of immune factors in rats with CP. Our data revealed that in the model group, the level of SIgA and the secretion of IgG in sera of rats with CP increased significantly. These changes were reversed in a dose-dependent manner after calycosin treatment, suggesting that calycosin can reduce the levels of immune factors in rats with CP.

CP can be alleviated via anti-inflammatory, antioxidant, and anti-fibrotic treatments. Oxidative stress is linked to the occurrence and progression of CP [22]. MDA is a product of lipid peroxidation that participates in oxidative stress-induced injury. SOD and CAT scavenge free radicals to protect cells from oxidative stress damage [23]. Damage to epithelial cells may result in the release of inflammatory cells and ROS [24]. Based on previous studies, we determined ROS, MDA, SOD, and CAT levels to investigate whether calycosin plays a role in oxidative stress in CP. We observed increased ROS release in the prostate tissue by immunofluorescence, higher MDA levels, and lower SOD and CAT activities than in the control group, which is similar to the results of Jahan et al. [25]. However, we observed the opposite results in a dose-dependent manner in the calycosin-treated group, indicating that calycosin may protect against oxidative stress injury.

p38MAPK is one of the main members of the MAPK family that participates in the regulation of inflammatory responses [26]. NF-κB is a downstream gene involved in cell apoptosis, proliferation, and inflammatory reaction [27]. The p38 MAPK has been reported to be involved in NF-κB signal pathway activation [28]. Including TNF-α, IL-1β, and IL-6 inflammatory cytokines can activate the p38MAPK pathway, and the activated p38MAPK also plays a key role in regulating the biosynthesis and transcription of pro-inflammatory cytokines [29]. Oxidative stress can activate MAPK/NF-κB pathway. p38 MAPK/NF-κB is an ROS-sensitive signaling pathway, and ROS can activate the MAPK pathways, further stimulating several inflammatory cytokines [30]. In addition, multiple studies have also reported that the p38 MAPK/NF-κB signaling pathway plays an important role in the activity of antioxidant enzymes (SOD and CAT) and the production of MDA [31,32]. Therefore, the inhibition of inflammation and oxidative stress by adjusting the p38MAPK/NF-κB signal pathway may be an effective way to treat CP. Our findings suggested that calycosin inhibited the expression of p-p38 and p-p65 in the prostate tissue of CP rats in a dose-dependent manner, revealing that calycosin protects against CP by regulating the p38MAPK/NF-κB pathway.

Taken together, our observations demonstrated that calycosin can relieve inflammatory response by inhibiting the p38MAPK/NF-κB signaling pathway in CP, providing a basis for the prevention and therapy of CP with calycosin.
